# Arbuscular mycorrhiza can be disadvantageous for weedy annuals in competition with paired perennial plants

**DOI:** 10.1038/s41598-022-24669-6

**Published:** 2022-12-01

**Authors:** Veronika Řezáčová, Milan Řezáč, Gail W. T. Wilson, Tereza Michalová

**Affiliations:** 1 Institute of Microbiology, Czech Academy of Sciences, Vídeňská 1083, Prague 4, Czech Republic; 2 Crop Research Institute, Drnovská 507, Prague 6, Czech Republic; 3 Department of Natural Resource Ecology and Management, Oklahoma State University, Stillwater, OK USA

**Keywords:** Ecology, Microbiology

## Abstract

Arbuscular mycorrhizal (AM) fungi can support the establishment of mycotrophic plants in new environments. However, the role of mycorrhizal symbiosis in interactions between perennial and weedy annual plants is not well understood. In our current study, we examine how widespread generalist AM fungi and soil disturbance, including disturbance of AM fungal networks (CMNs), affect the performance of two late-successional perennial plants of Central Europe, *Senecio jacobaea* and *Crepis biennis,* co-occurring with weedy annual forbs, *Conyza canadensis* and *Erigeron annuus*. Although presence of weedy annual *E. annuus* or *C. canadensis* did not affect the performance of the paired perennials, AM fungi supported perennial *C. biennis* in competition with weedy annual *E. annuus*. However, this AM-aided underpinning was independent of disturbance of CMNs. Conversely, although AM fungi benefited perennial *S. jacobaea*, this did not affect its competitive abilities when grown with weedy annual *C. canadensis*. Similarly, soil disturbance, independent of AM fungal presence, improved plant tissue P and biomass production of *S. jacobaea*, but not its competitive abilities. Our results show AM fungi may be advantageous for perennial plants growing in competition with weedy annual plants. Therefore, maintaining healthy soils containing an abundance of AM fungi, may encourage late successional perennial plants, potentially limiting establishment of weedy annual plant species.

## Introduction

The majority (> 60%) of land plants^1,2^, including most annual plant species^3–5^, form mycorrhizal interactions with arbuscular mycorrhizal (AM) fungi (Glomeromycotina;^6^). AM fungi supply host plants with nutrients (especially phosphorus (P)^7^) from the soil, aid in protecting against plant pathogens^8,9^, and potentially an array of other services in exchange for carbon from the host plant^10,11^. For example, the AM fungus *Gigaspora margarita*, increased P uptake of maize by 55%^12^. Further, under P-limiting soil conditions, AM fungi can be responsible for nearly all P uptake by the mycorrhizal host plant^13,14^. These strong benefits often result in AM fungi affecting coexistence of plant species, and therefore the composition of plant communities^15–17^. Therefore, these symbiotic fungi may enhance or prevent establishment of weedy annuals, or increase the competitive abilities, and thereby persistence, of perennial species.

Both annual and perennial plant species generally form an association with AM fungi for competitive success^1–5^. Likewise, AM fungal symbionts may be essential for naturalization and spreading of weedy annual plants, yet this has rarely been studied (although see^18–20^). Weedy plant species are generally annuals representing the R strategy, and are typically facultative mycorrhizal^21,22^. Facultative mycorrhizal plants form weak associations with AM fungi, can complete their life cycle in the absence of the symbiosis, and are generally characterized by low levels of AM fungal root colonization^21,22,24^. The annual life history strategy and facultative AM association can be potentially beneficial in disturbed habitats where intact AM fungal communities and competition for resources are low. Perennials, in contrast, develop long-term life history strategies for competition for limited resources, as they survive for extended periods of time. Short-term strategies of annuals may be disadvantageous in established plant communities where hyphal networks connect individual perennial plants and transfer nutrients, mainly N and P, between plants (i.e., common mycorrhizal networks: CMNs^23^). Commandeering these networks can increase competitiveness of some plants, allowing disproportionately greater benefits compared to symbiotic carbon costs.

Life-history strategies are generally indicative of AM benefit, with late-successional perennial plants, established in undisturbed stable systems, receiving greater benefit than early successional species that colonize disturbed or unstable sites. In fact, AM fungal inoculation has been shown to increase competitive ability of perennials when in competition with annuals^24^. However, the role of mycorrhizal symbiosis in interactions between perennial and weedy annual plants following disturbance is not well understood, and information detailing the influence of AM fungi in the competitive success of these different life history strategies is not clear.

However, at ecosystem level, it has been shown that AM fungi are often susceptible to perturbations, such as soil disturbance^25–27^, as these disturbances disrupt CMNs^28^. The subsequent recovery of CMNs is costly for both fungi and host plants, and thus may delay potential mycorrhizal advantages provided to the host plant. For example, disrupted CMNs may delay or limit the competitive advantage of a perennial plant, promoting at least a temporary advantage to a weedy annual plant, as the facultatively mycotrophic annual is not required to receive benefits from AM fungi. Alternatively, the perennial plant, dependent on the symbiosis, may lose the competitive advantage, at least until CMNs are re-established. However, the effects of CMNs disturbance on competition between plants with different life history strategies, such as perennial and annual species, in not known. Understanding the role of disturbed CMNs in providing advantages to weedy annuals may be critical to restoration or rehabilitation of disturbed lands.

In our current study, we focused on competition of two important weedy annual plant species paired with co-occurring perennial forbs. Our study was conducted using widespread generalist AM fungi to reduce the possibilities of host species advantage. We selected two perennial plant species, *Senecio jacobaea* and *Crepis biennis* (mycotrophic and native to Central Europe), and two weedy annuals, *Conyza canadensis* and *Erigeron annuus* (facultatively mycotrophic and invasive species in Central Europe), to assess if CMNs and an initial disturbance affect their performance.

We hypothesized that (i) CMNs increases the success of a perennial plant in competition with a weedy annual plant; and (ii) the competitive disadvantage of the weedy annual plant against the perennial is more pronounced in soil with intact (non-disturbed) CMNs, as compared to initially disturbed CMNs and; (iii) the observed negative effect increases with time.

## Results

### Competition for resources between annual and perennial plants

**Figure 1:**
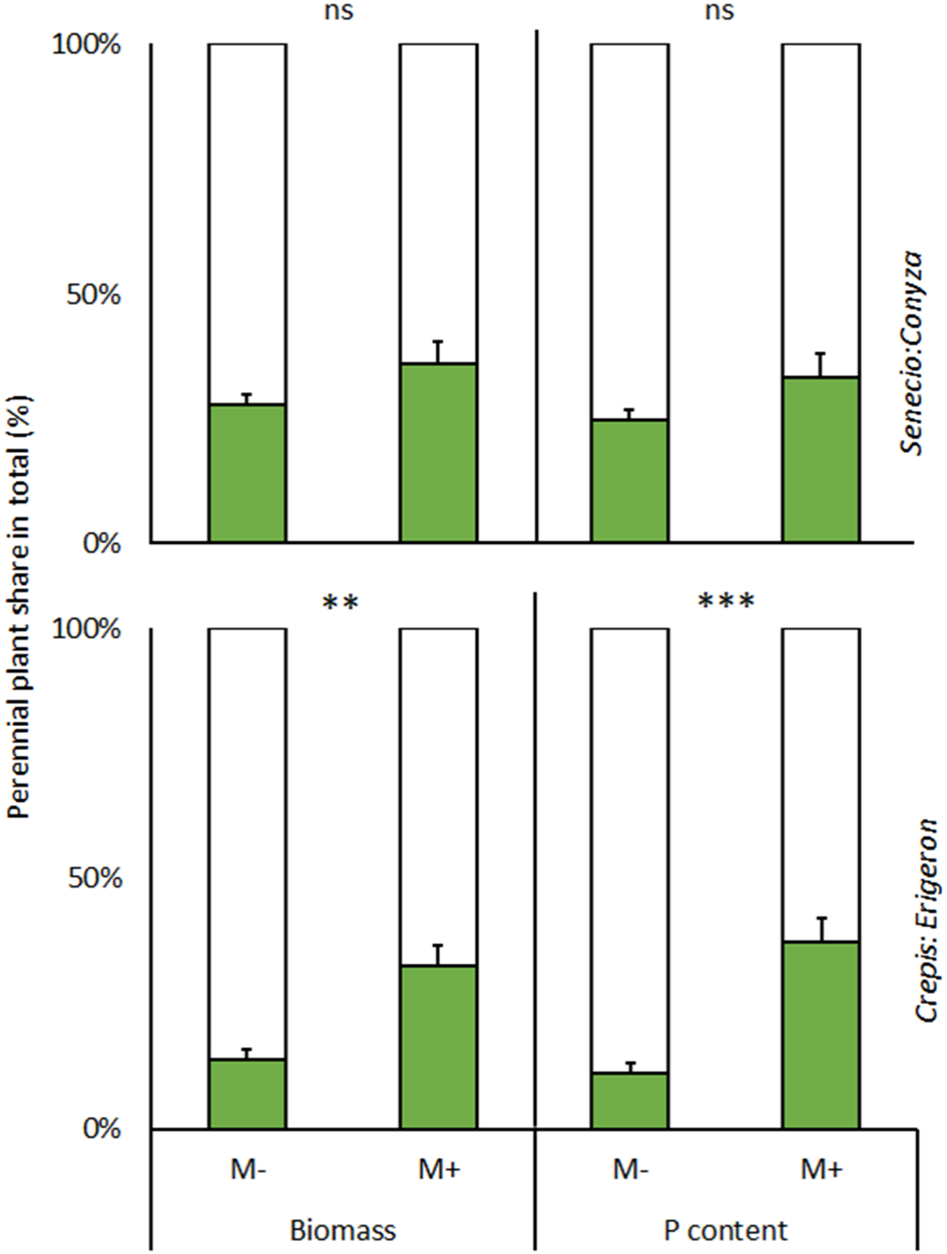
Fraction of shoot dry biomass and phosphorus content of perennial plants (gray bars) paired with weedy annual plants (white bars) as follows: *Conyza canadensis-Senecio jacobaea* (*Senecio:Conyza*) and *Erigeron annuus–Crepis biennis* (*Crepis:Erigeron*), determined by plant biomass at each cultivation (i.e., the share of resources acquired by the perennial plant on a whole cultivation basis, with the remainder assignable to the weedy annual plant) as affected by mycorrhizal inoculation (M+: mycorrhizal inoculum added; M−: nonmycorrhizal control). Bars represent means accompanied by standard errors (n = 10).

Relative *C. biennis* biomass and P content were significantly correlated (P ≤ 0.05) and were both greater in M+ than in M– plants (Fig. 1) independent of disturbance (Table 1). However, in the case of *S. jacobaea*, mycorrhizal inoculation and disturbance had no effect on relative biomass production or relative P acquisition (Table 1, Fig. 1). The results of harvest 1 (not shown) were not different from those of harvest 2.

**Table 1:** Significance of effects of mycorrhizal inoculation and disturbance, and their interaction on perennial plants *Senecio jacobaea* and *Crepis biennis* contribution to total shoot dry biomass and total shoot phosphorus contents.

		*S. jacoabea*		*C. biennis*	
		Biomass	Phosphorus	Biomass	Phosphorus
Inoculation	*P*	0.39	0.41	**3.72 × 10**^**–3**^	**5.90 × 10**^**–4**^
	*F*_*1.16*_	0.8	0.7	11.8	18.8
Disturbance	*P*	0.60	0.47	0.45	0.38
	*F*_*1.16*_	0.3	0.5	0.6	0.8
Inoculation × disturbance	*P*	0.53	0.64	0.99	0.98
	*F*_*1.16*_	0.4	0.2	0.0	0.0

*F*‐values (F) and levels of statistical significance (*p*-value ranges, *P*) as per two-way ANOVAs are shown (n = 5).

Significant results are stated in bold.

### Effects of weedy annuals on plant biomass and mineral nutrition of perennial plants

**Figure 2:**
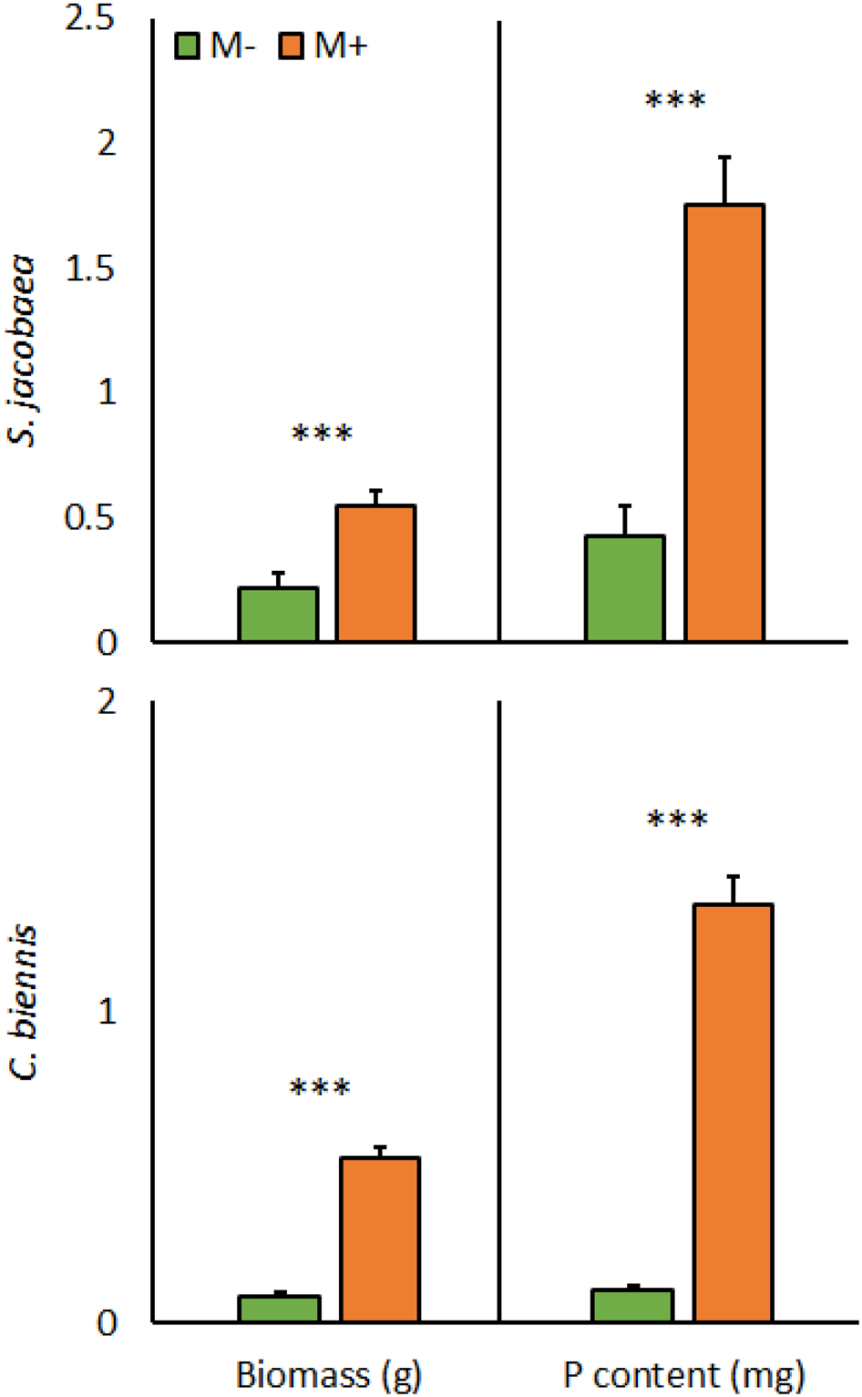
Shoot biomass and phosphorus content of perennial plant species *Senecio jacobaea* and *Crepis biennis* either paired with *C. canadensis* and *E. annuus*, respectively, or not, as affected by mycorrhizal inoculation (M+: mycorrhizal inoculum added; M−: nonmycorrhizal control). Bars represent means accompanied by standard errors (n = 20).

The biomass and plant P content of both perennials, *C. biennis* and *S. jacobaea*, were increased by mycorrhizal inoculation (Table 2, Fig. 2), and P content was positively correlated with biomass (P ≤ 0.05). Disturbance significantly increased P content (non-disturbed pots 0.85 ± 0.17 mg, disturbed pots 1.35 ± 0.24 mg) and biomass (non-disturbed pots 0.30 ± 0.05 g, disturbed pots 0.48 ± 0.08 g) of *S. jacobaea* but had no effect on these parameters for *C. biennis* (Table 2). Plants grown in monocrop or multicrop did not affect plant biomass or P content of either *S. jacobaea* or *C. biennis* (Table 2). The results of harvest 1 (not shown) were not different from those of harvest 2.

**Table 2:** Significance of effects of mycorrhizal inoculation, plant community composition (monocrop or multicrop) and disturbance, and their interaction on shoot dry biomass and shoot phosphorus content of perennial plants *Senecio jacobaea* and *Crepis biennis. *

		*S. jacoabea*		*C. biennis*	
		Biomass	Phosphorus	Biomass	Phosphorus
Inoculation	*P*	**5.21 × 10**^**–4**^	**4.29 × 10**^**–6**^	**2.13 × 10**^**–13**^	**3.04 × 10**^**–14**^
	*F*_*1.32*_	15.0	30.9	144.3	167.0
Plant community composition	*P*	0.83	0.51	0.13	0.41
	*F*_*1.32*_	0.0	0.4	2.4	0.7
Disturbance	*P*	**4.18 × 10**^**–2**^	**4.84 × 10**^**–2**^	0.60	0.99
	*F*_*1.32*_	4.5	4.2	0.3	0.0
Inoculation × plant community composition	*P*	0.33	0.62	0.62	0.86
	*F*_*1.32*_	1.0	0.3	0.3	.0
Inoculation × disturbance	*P*	0.98	0.63	0.99	0.91
	*F*_*1.32*_	0.0	0.2	0.0	0.0
Plant community composition × disturbance	*P*	0.98	0.98	0.72	0.64
	*F*_*1.32*_	0.0	0.0	0.1	0.2
Inoculation × disturbance × plant community composition	*P*	0.87	0.86	0.64	0.93
	*F*_*1.32*_	0.0	0.0	0.2	0.0

*F*‐values (F) and levels of statistical significance (*p*-value ranges, *P*) as per three-way ANOVAs are shown (n = 5).

Significant results are stated in bold.

### AM fungal development

No roots of M− plants contained AM fungal structures, while AM fungi were present in roots of all M+ plants (see Table S4). Although microscopic assessment of AM fungal abundance in roots was performed only in M− plants, a control sample of M+ plants intended for determination of AM fungal colonization determined plant roots were highly colonized by AM fungi (Table S3).

**Figure 3:**
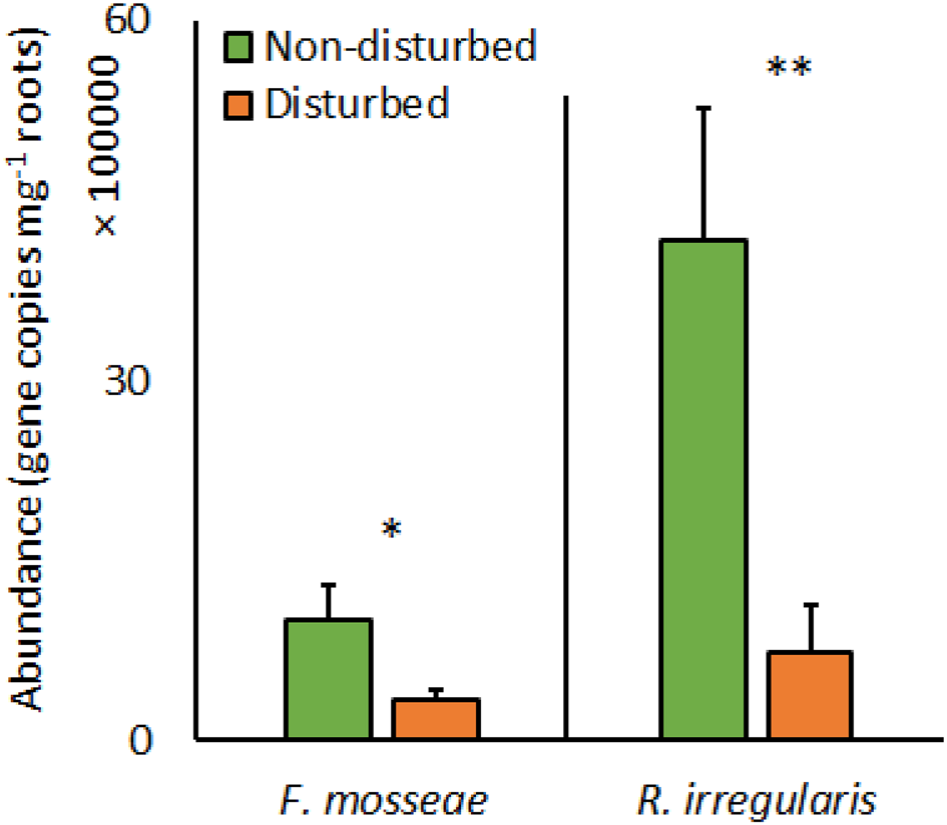
Abundance of arbuscular mycorrhizal fungal taxa in roots of the perennial plant species *Crepis biennis* either paired or not with weedy annual, *Erigeron annuus*, respectively, as affected by soil disturbance. Bars represent means accompanied by standard errors (n = 10).

Plant growth in monocrop or multicrop did not significantly affect the abundance of any AM fungal taxa in roots of *S. jacobaea* or *C. biennis* (Table 3). Disturbance tended to decrease the abundances of *R. irregularis* and *F. mosseae* (*C. claroideum* was not present in roots of any plant of harvest 2) in the roots of *C. biennis* (Table 3, Fig. 3). However, disturbance had no effect on AM fungal abundances in roots of *S. jacobaea* (Table 3).

**Table 3:** Significance of effects of plant community composition (monocrop or multicrop) and disturbance, and their interaction on the abundance of different arbuscular mycorrhizal fungal taxa in root samples of the perennial plant species *Senecio jacobaea* and *Crepis biennis*.

		*S. jacoabea*		*C. biennis*	
		*F. mosseae*	*R. irregularis*	*F. mosseae*	*R. irregularis*
Plant community composition	*P*	0.13	0.76	0.13	0.24
	*F*_*1.16*_	2.5	0.1	2.6	1.5
Disturbance	*P*	0.23	0.14	**0.04**	**0.01**
	*F*_*1.16*_	3.1	7.1	0.8	3.3
Disturbance × plant community composition	*P*	0.94	0.07	0.11	0.06
	*F*_*1.16*_	0.0	3.8	2.9	4.0

*F*‐values (F) and levels of statistical significance (*p*-value ranges, *P*) as per two-way ANOVAs are shown (n = 5).

Significant results are stated in bold.

**Figure 4:**
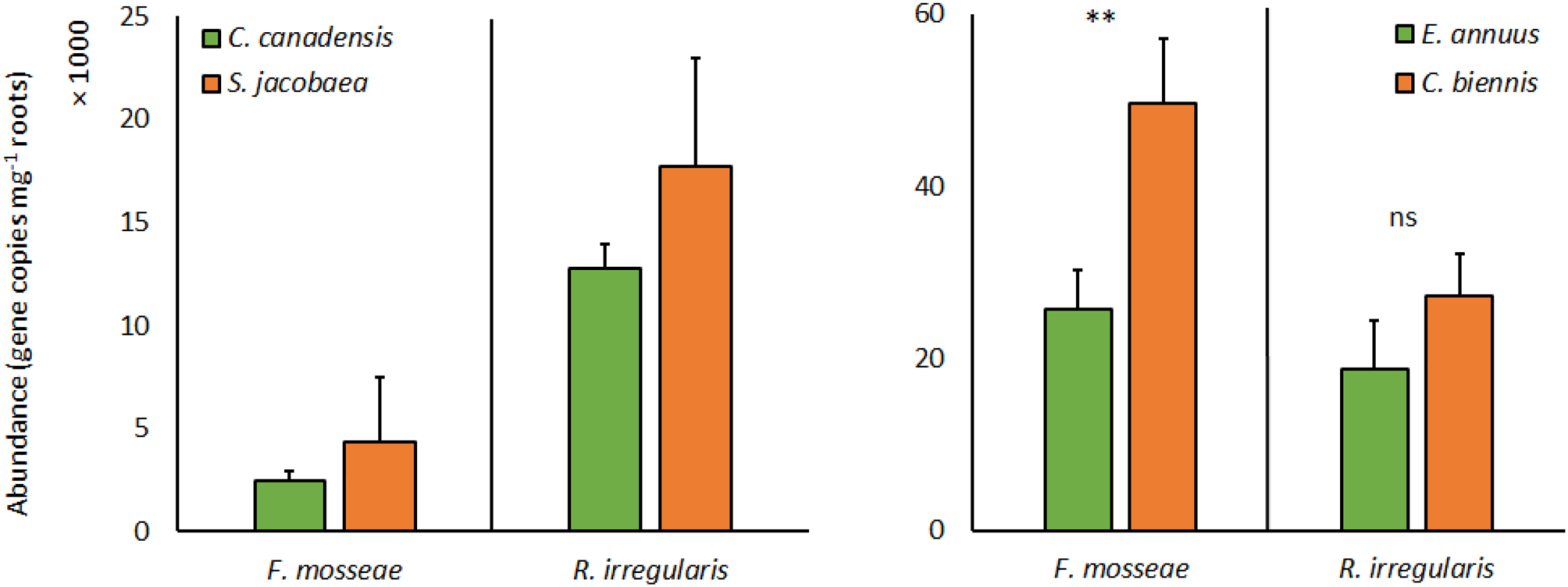
Abundance of arbuscular mycorrhizal fungal taxa in roots of the perennial plant species, *Senecio jacobaea* and *Crepis biennis*, and paired weedy annual plants, *Conyza canadensis* and *Erigeron annuus*, respectively, co-occurring in the experimental pots as affected by plant species. Bars represent means accompanied by standard errors (n = 10).

The abundances of individual AM fungal taxa were not significantly different between roots of weedy annual plant *C. canadensis* or its paired perennial plant *S. jacobaea* (Table 4, Fig. 4). The abundances of *R. irregularis* did not differ between weedy annual plant *E. annuus* roots and paired perennial *C. biennis*. However, *E. annuus* was colonized by lower abundances of *F. mosseae*, compared to paired *C. biennis* (Table 4, Fig. 4). Whereas disturbance equally increased the abundance of *F. mosseae* of both species, *E. annuus and C. biennis*,grown in competition (non-disturbed pots 30 ± 6 × 10^5^ CN mg^−1^ roots, disturbed pots 45 ± 6 × 10^5^ CN mg^−1^ roots) (Table 4). The results of harvest 1 (not shown) were not different from those of harvest 2.

**Table 4:** Significance of effects of plant and disturbance, and their interaction on the abundance of different arbuscular mycorrhizal fungal taxa in root samples of the perennial and annual plant species co-occurring in the experimental pots.

		*C. canadensis–S. jacoabea*		*E. annuus–C. Biennis*	
		*F. mosseae*	*R. irregularis*	*F. mosseae*	*R. irregularis*
Plant species	*P*	0.16	0.43	**3.80 × 10**^**–3**^	0.24
	*F*_*1.16*_	2.2	0.6	11.4	1.5
Disturbance	*P*	0.20	0.09	**4.66 × 10**^**–2**^	0.23
	*F*_*1.16*_	1.8	3.4	4.7	1.5
Disturbance × plant species	*P*	0.97	0.90	0.54	0.46
	*F*_*1.16*_	0.0	0.0	0.4	0.6

*F*‐values (F) and levels of statistical significance (*p*-value ranges, *P*) as per two-way ANOVAs are shown (n = 5).

Significant results are stated in bold.

## Discussion

We observed positive effects of mycorrhiza on the biomass and P acquisition of both perennial plants assessed in our study, and this was demonstrated positively or neutrally when perennial plants were grown in competition with the respective weedy annual. This agrees with our hypothesis that perennial plants will be more supported by mycorrhiza, and therefore have greater success in competition with the facultatively mycotrophic weedy annual plants and partially agrees with a review by Lin et al.^24^. However, our findings are not similar to Callaway et al.^19^ or Řezáčová et al.^29^, as these reported AM fungi supported weedy annuals (*Centaurea melitensis* or *E. sphaerocephalus*, respectively), when grown in competition with a perennial plant. Our current study agrees with Callaway et al.^30^, in that the influence of AM symbiosis on competitive abilities of weedy or invasive plants are context-dependent and should not be generalized, highlighting the importance of assessing influences of AM fungi on specific plant species.

Although AM fungal inoculation played a positive role on perennial plant performance in the competition between perennial plant *C. biennis* and weedy annual plant *E. annuus*, the influence of the inoculation was nevertheless independent of the initial disturbance. Thus, it was not possible to confirm the role of intact CMNs unevenly redistributing nutrients in the competition of these plants with differing life-history strategies.

### The role of AM fungi in competition between perennial plant S. jacobaea and weedy annual plant C. canadensis

*C. canadensis* is an important weedy annual, expanding throughout introduced, as well as native, areas. The rapid global expansion of *C. canadensis* has been attributed to a combination of factors. For example, effective reproduction and effective utilization of newly created habitats are likely mechanisms promoting *C. canadensis* expansion (e.g.,^31^). Despite having an annual life strategy, association with AM fungi has also been hypothesized as a mechanism for successful expansion of *C. canadensis*^32–34^. In agreement with other research^35–38^, *C. canadensis* formed associations with AM fungi in our current study. However, the lack of any negative effect of *C. canadensis* presence on performance of *S. jacobaea* in our study does not support the possibility that AM fungi could contribute to *C. canadensis* spread; the coexistence of these two plants was independent of AM symbiosis in our experiment. While AM symbiosis was beneficial for P-uptake and biomass production of *S. jacobaea*, the symbiosis did not increase the plant performance of *S. jacobaea* when grown with *C. canadensis*, presumably because AM fungi were beneficial for both plant species in our study. A previous study, contrary to our results, reported negative responses of perennial *Anthemis cotula* on neighbouring *C. canadensis* were mediated by AM fungi^36^. Therefore, it should be cautioned that mycorrhizal effects between neighboring plants of different species is likely context-dependent, as our findings showed no influence from mycorrhiza in the competition of *C. canadensis* with the perennial plant *S. jacobaea*.

### The role of AM fungi in competition between weedy annual plant E. annuus and perennial plant C. biennis

*Erigeron annuus* is another example of a weedy annual plant successfully increasing in abundance throughout Europe. The expansion of *E. annuus* has been credited to increased seed survival, rapid seedling and rosette development, and/or allelopathic activity^39^. Because *E. annuus* readily forms mycorrhizal symbiosis^40–42^, mechanisms allowing successful expansion of *E. annuus* could also include AM symbiosis. However, based on our results, we cannot confirm AM fungi as a mechanism used by this annual plant when in competition with the perennial *C.* *biennis*. This is partly supported by Rinauldo et al.^43^ and Veiga et al.^44^ who found weeds grown in community to be negatively influenced by AM fungi. In fact, in our current study, AM fungi increased the relative biomass and P content of perennial *C. biennis* when paired with *E. annuus*. As we hypothesized, the greater mycorrhizal responsiveness of perennial plants compared to annual plants^45^ play a role in the higher responsiveness of perennial *C. biennis* on AM fungal inoculation, compared to annual *E. annuus*. Based on our results, we suggest the more responsive perennial *C. biennis* is more competitive in soils with a natural occurrence of AM fungi, when paired with *E. annuus*.

The mycorrhizal benefit of perennial *C. biennis* was nevertheless independent of the initial soil disturbance even though the disturbance decreased the abundance of AM fungi in their roots. Therefore, although *C. biennis* and *E. annuus* were associated with similar AM fungal taxa with the potential for resource sharing through intact CMNs in non-disturbed soil, this was not likely a major mechanism driving the positive effects of AM fungi or promoting greater competitive performance of *C. biennis* over the annual *E. annuus*. Alternatively, it is likely the disturbed CMNs recovered rapidly following disturbance. Rapid recovery of CMNs after disturbance would lead to similar results between disturbed and intact CMNs.

### The effect of a single soil disturbance on competition of perennial and weedy annual plants

Both plant tissue P content and biomass production of perennial *S. jacobaea* increased following disturbance. However, the relative performance of *S. jacobaea*, when grown with co-occurring weedy annual plant *C. canadensis*, was not affected by disturbance, as the performance of *C. canadensis* increased to the same extent as *S. jacobaea*. Soil disturbance likely increased soil aeration and microbial activity, temporarily increasing P-availability and subsequent P supply to both co-occurring plants^46^. Furthermore, mycorrhizal plants did not differ from nonmycorrhizal plants, in either undisturbed or disturbed soils, indicating host plant reliance on AM fungi for P-uptake was apparently not substantial under these conditions.

Although increases in nutrient availability as a result soil disturbance has been previously reported^46,47^, in our study initial soil disturbance did not affect performance of nonmycorrhizal *C. biennis*; increased P availability caused by the disturbance was not reflected as increased P uptake by the plant. Because P availability for both AM inoculated plants and non-inoculated plants in both disturbed and undisturbed treatments was constant, increased P supply would be beneficial for all plants if P-availability was limited, and any increase in P uptake would not be considered luxurious. In fact, in our study, P did not appear to be limiting, as presence of weedy annual plant *E. annuus* did not affect the P content in *C. biennis* shoots. However, although the overall system was not P limiting, P could be limiting for individual plants when in competition with a neighboring plant more efficient at P-uptake, which likely occurred when perennial *C. biennis* acquired less P compared to corresponding perennial *S. jacobaea* when grown under the same conditions. Reduced P-uptake by *C. biennis* may be because another factor that we did not measure was more limiting for this species.

Disturbance did not influence the performance of perennial *C. biennis* or the co-occurring weedy annual plant *E. annuus*. Contrary to our hypothesis, based on the ruderal nature of weedy annual plants, the single soil disturbance did not play a role in the performance of weedy annual plant *E. annuus* when grown in competition with perennial plant *C. biennis*, or *C. canadensis* when grown in competition with perennial *S. jacobaea*. In a previous study, a single soil disturbance did not influence performance of *Inula conyzae*, a native plant species when grown in competition with *Echinops sphaerocephalus*, a non-native invasive plant species common in Eastern Europe^29^, and the authors propose the lack of response to disturbance was likely due to a rapid recovery of CMNs following disturbance. We propose a similar rapid recovery of the CMNs occurred in our current study, and therefore, plants with intact CMNs were not at an advantage over plants with initially disturbed mycelium.

In our study, P was not limiting, and a single soil disturbance did not affect AM fungi or alter AM benefits for either the perennial or the weedy annual plants. This indicates CMNs following a single soil disturbance will likely recover quickly and, therefore, not support the performance and subsequent expansion of weedy plants species as we initially hypothesized. However, we caution that mycorrhizal fungi may be considerably more important under other soil disturbances, especially long-term disturbances such as drought, salinity, soil compaction, or nutritional stress^48,49^.

## Conclusions

Our data partly support the hypothesis that AM fungi enhances the competitive abilities of the perennial plants, *S. jacobea* and *C. biennis*, against weedy annual plants, *C. canadensis* and *E. annuus*. Specifically, AM fungi benefited perennial *C. biennis* when grown with the weedy *E. annus*, but did not benefit the performance of perennial *S. jacobea* when paired with the weedy annual plant *C. canadiensis*, although AM fungi did increase the performance of both these species. Based on our results, it is not possible to attribute effects of AM fungi to the uneven distribution of nutrients by intact CMNs, as soil disturbance did not influence performance of either of the two weedy annuals when grown with corresponding perennials, although the success of many weedy annual plant species is facilitated by habitat disturbance^50^. In fact, the single soil disturbance of our study supported biomass production and P acquisition of perennial *S. jacobaea*, independent of the paired plant species.

Although our controlled greenhouse experiment was limited to pair-wise interactions among plant species, this is a critical first step in determining complex interactions that occur among perennial and weedy annual plant species following a single soil disturbance. Although our study assessed only two pairs of plant species, the data indicate it is critical to maintain the natural occurrence of AM fungal communities to prevent the spread of weedy, including non-native invasive, plant species, and reducing soil disturbance through sustainable and low-impact management may reduce the expansion of invasive plant species. However, while our study provides important baseline information, to support the practical and ecological relevance of our findings further research, especially studies conducted under field conditions, should be encouraged.

## Materials and methods

### Experimental design

**Figure 5:**
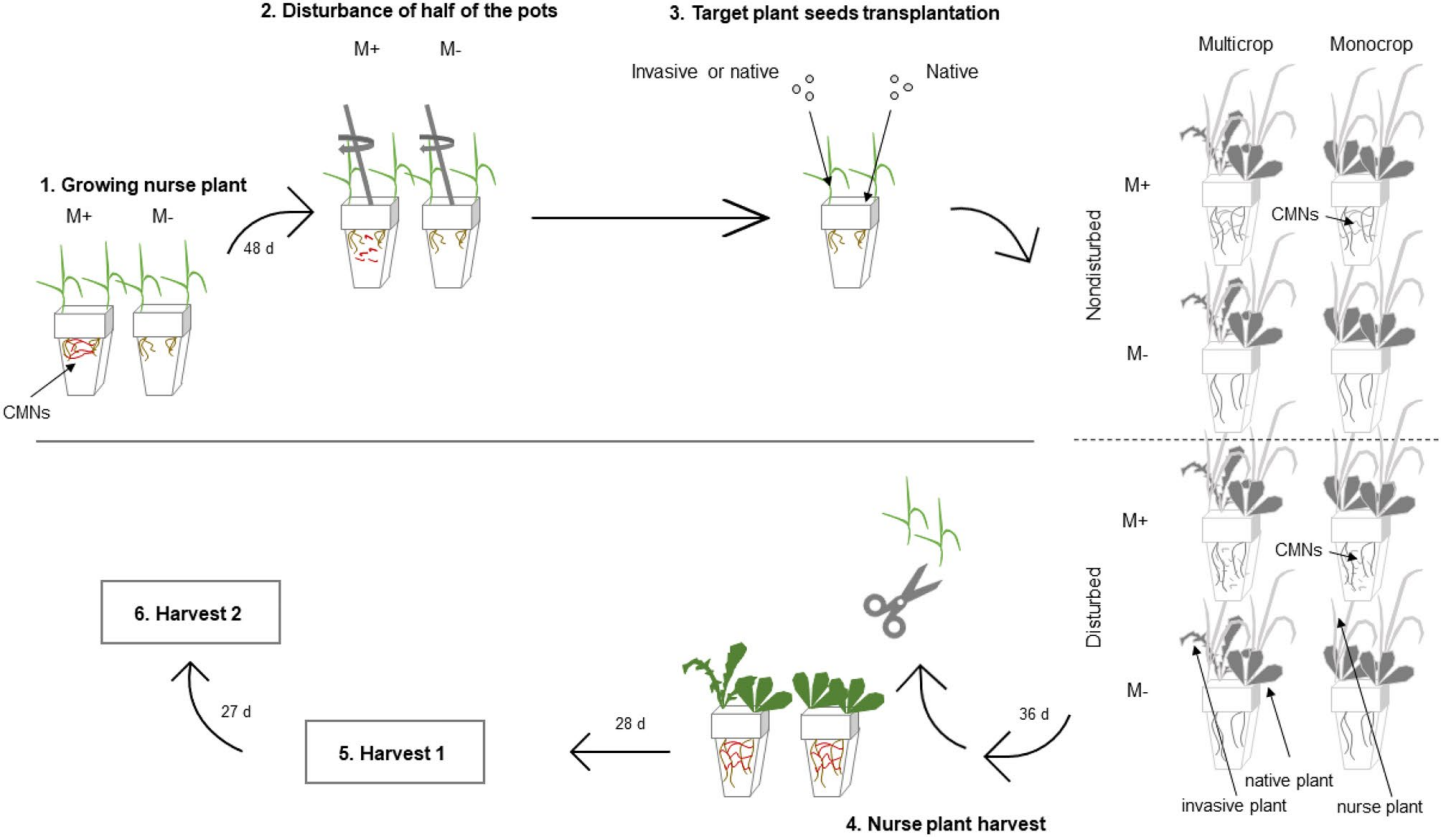
Procedural flow diagram showing individual steps of the experiment at the time frame and integrating experimental design. This design was used for both weedy annual-perennial plant species combinations, and both harvests. Mycorrhizal (M+) and nonmycorrhizal (M−) pots were pre-planted with *Festuca pratensis* as a nurse plant. Soil was disturbed or remained intact prior to annual and perennial plants being planted, resulting in disturbed or non-disturbed common mycorrhizal networks (CMNs) in the M+ treatments. Each figured pot contained 5 replicates (for each harvest and each perennial-weedy-annual plant combination). Figure modified from Řezáčová et al.^43^.

We conducted a greenhouse experiment to study pairwise interactions between mycorrhizal weedy annual and perennial plants (weedy annual—perennial: *Conyza canadensis—Senecio jacobaea; Erigeron annuus—Crepis biennis*). *Erigeron annuus* (L.) Pers. and *Conyza canadensis* (L.) Cronq., are common annual weedy plant species of seminatural plant communities in Central Europe (Czechia). The corresponding perennial species, *Crepis biennis* L.; and *Senecio jacobaea* L., are from the same plant family (Asteraceae) as the weedy annual plant species and occur abundantly in *E. annuus* or *C. canadensis* inhabited plant communities. We imposed two mycorrhizal treatments: inoculated with mycorrhiza (M+) or not (M-), combined with a disturbance treatment: soil initially mechanically disturbed or not (leading to intact or disturbed CMNs in M+ pots). Biomass production of perennial plants in the presence of weedy annual plants were assessed for perennial plants in monocrops (perennial paired with perennial) or multicrops (perennial paired with weedy annual). Competition between the perennial and weedy annual plant was evaluated at two experimental harvest times [64 days (harvest 1) or 91 days (harvest 2)] after plant transplantation. For the experimental design see Fig. 5. Each treatment combination contained five replicate pots with a total of 160 pots set up at the beginning of the experiment (see Fig. 5). The positions of the pots in the greenhouse were randomized.

### Cultivation pots and substrate

Similar to Řezáčová et al.^29^, plants were grown in 2-L pots (11 × 11 × 20 cm, w × d × h) filled with a substrate comprised of 10:45:45 mixture of 10% γ-irradiated (> 25 kGy) Central European field soil from Litoměřice, Czechia (N50°31′54.53′′ E14°06′7.10′′), 45% autoclaved zeolite MPZ 1–25 from Zeopol (https://www.zeolity.cz, grain size 1–2.5 mm), and 45% autoclaved quartz sand (grain size < 3 mm). Physicochemical properties of the substrate are detailed in Řezáčová et al.^51^ or Table S1.

### Mycorrhizal inoculation

Mycorrhizal pots (M+) were inoculated with 36 g of AM fungal inoculum consisting of potting substrate containing root fragments of leek (*Allium porrum* L.), used as the host plant to develop pot cultures of AM fungal isolates, all widespread generalists of Central European origin. Inoculum in our current study contained the following AM fugal taxa: *Claroideoglomus claroideum* (N. C. Schenck& G. S. Sm.) C. Walker & Schuessler (2010) BEG 155, *Funneliformis mosseae* (T.H. Nicolson & Gerd.) C. Walker & Schuessler (2010) BEG 161 and *Rhizophagus irregularis* (N.C. Schenck & G.S. Sm.) C. Walker & Schuessler (2010) BEG 158 (https://www.i-beg.eu). These monospecific inocula were mixed as a volume ratio 1:1:1 (*v:v:v*) to create a synthetic community of widespread generalist AM fungi, resulting in 492 spores of. *C. claroideum*, 588 spores of *F. mosseae* and 612 spores of *R. irregularis* added to each pot. Nonmycorrhizal (M–) pots received 36 g non-mycorrhizal (mock) inoculum, which was added 4–5 cm below soil surface of each pot. The mock inoculum contained substrate and leek root fragments from cultures grown under the same conditions, for the same amount of time, as the M+ pot cultures (above), but without AM fungi, as assessed microscopically.

To promote the growth of CMNs in the M+ plots, we used *Festuca pratensis* Huds. as a nurse plant, which was also used in the M− pots to control for nutrient depletion (Fig. 5). Seeds of *F. pratensis* were directly sown into each pot in the middle of January. After 48 days, substrate (and CMNs in M+ pots) in half of both M+ and M− pots were mechanically disrupted as follows: a long metallic spatula was inserted to the full depth of the pot, and wide circular movements progressing from one corner of the pot to the other, disturbing the entire volume of the pot (Fig. 5). Microscopic assessment of soil samples (according to Newman^52^) of 6 M+ determined hyphal length densities and these densities were similar to a previous experiment^53^.

### Plant cultivation and preparation

The seeds of all the experimental plants were field collected in the Czech Republic. Our study complies with relevant institutional, national, and international guidelines and legislation on handling plant material.

Seeds were germinated for two weeks at room temperature on wet filter paper contained in Petri dishes. One of the empty corners of the pot was planted with one perennial species, whereas the opposite corner was planted with perennial or weedy annual plant species (see Fig. 5). Note that sowing was conducted immediately after soil disturbance simulations. Plants in M+ treatments were planted into non-disturbed soil with intact CMNs, or into initially disturbed soil where CMNs needed to reestablish from spores and hyphal fragments. Thirty-six days following the initial disturbance, shoots of all nurse plants were harvested, dried, and weighed. Nurse plants did not re-grow after harvest and there were no significant differences between nurse plant biomass grown in multicrop or monocrop pots.

Annual and perennial plants were grown for an additional 28 days (harvest 1) or 55 days (harvest 2) (March–May) 2018 in a greenhouse at the Institute of Microbiology, Prague with average day and night temperatures of 24 °C and 20 °C, respectively. Day length was extended to 12 h using supplemental lighting. Temperature and daylength were selected to represent typical field conditions of Central Europe (March–May). Following nurse plant planting, each pot received 65 ml of Long Ashton mineral nutrient solution every week^54^ with the P concentration reduced to 20% of the original recipe^45,55^, to avoid constraint of AM fungal or plant growth due to P limitation.

### Plant harvest and analyses

The experiment was harvested twice to assess M+ annual and perennial plant production following initial disturbance. A harvest, plant shoots were cut at the hypocotyl–root interface and subsequently dried for 3 days at 65 °C to determine shoot dry weight. Substrate was thoroughly washed from roots, roots were weighed and cut into 1.5 cm fragments, and fragments were divided into three portions. One portion of the root fragments was immersed in 50% ethanol to determine AM root colonization. The second was stored in a freezer at −20 °C for molecular analyzes. The final portion was weighed, dried and reweighed, to determine root water content and root dry biomass.

Plant dry weight and tissue P content were assessed to evaluate benefits provided by AM fungi to each plant. P concentration in aboveground plant tissues was assessed as described in Řezáčová et al.^56^. Orthophosphate concentration of the extracts was measured using the malachite green method^57^. Shoot P content (hereafter referred as plant P content) was calculated from shoot nutrient concentration using total shoot dry biomass.

The composition of AM fungal communities in the roots was assessed using quantitative real-time PCR (qPCR) markers (mt5, moss, and clar) targeting sequence-specific motifs of *Rhizophagus*, *Funneliformis*, and *Claroideoglomus*, respectively, in the mitochondrial (*Rhizophagus*) or nuclear (*Funneliformis* and *Claroideoglomus*) large ribosomal subunit RNA genes as described previously^58^.

Presence of AM fungal root structures was assessed microscopically in all M– pots, using one composite root sample for each M– pot (following McGonigle et al.^59^) after staining the roots with trypan blue (^60^; with minor modifications as in Řezáčová et al.^51^).

### Statistical analyses

Analyses of variance (ANOVA) with *P* < 0.05 as the significance cutoff level were calculated in R 4.2.0 statistical environment (R Core Team, 2022, http://www.R-project.org/^61^) after data was assessed for conformity with ANOVA assumptions (i.e., normality and homogeneity of variances). The analyses were conducted independently for harvest 1 and 2. Data are presented as mean values and standard errors for each treatment combination.

To assess if AM fungal inoculation and soil disturbance altered the plant performance of perennial or co-occurring paired weedy annual plants, we conducted two-way ANOVAs with the following factors: mycorrhizal inoculation and soil disturbance on perennial plant share in multicrops. Measures of P and biomass estimate the perennial plant share as the fraction of total plant biomass or plant P content that is the contributed to the perennial plant in each multicrop^29,57^.

To assess if plant growth in monocrop or multicrop, and disturbance, affect mycorrhizal shoot P content and biomass of the perennial plants, we used three-way ANOVAs with the following factors: mycorrhizal inoculation, soil disturbance and plant community composition (monocrop or multicrop). Plant community composition (monocrop or multicrop) was assessed to determine biomass and plant P content of each perennial plant species growing in monocrops or growing with the respective paired weedy annual plant.

To assess if the weedy annual plants and soil disturbance affect AM fungal abundances in the paired perennial plants, we performed two-way ANOVAs with the following factors: plant community composition (monocrop or multicrop) and soil disturbance on AM fungal taxon abundances of the perennial plant species growing in monocrops and multicrops. Additionally, to assess if the AM fungal taxon abundances differed between co-occurring perennial and weedy annual plants, and if this was affected by disturbance, we conducted two-way ANOVAs with the factors: plant species and soil disturbance on abundance of AM fungal taxa of each perennial plant species and paired weedy annual plants grown in multicrops.

## Supplementary Information

Supplementary Tables.
