# Supplementary material for: Arbuscular mycorrhiza can be disadvantageous for weedy annuals in competition with paired perennial plants
Source: Sci Rep. 2022 Dec 1;12:20703. doi: 10.1038/s41598-022-24669-6 (PMC9715701; doi:10.1038/s41598-022-24669-6)
Supplement: Supplementary file 1 — Supplementary Tables. [file 41598_2022_24669_MOESM1_ESM.pdf]

Supplementary Material

Arbuscular mycorrhiza can be disadvantageous for weedy annuals in competition with paired perennial plants

Veronika Řezáčová[1,2,*], Milan Řezáč[1,2], Gail W. T. Wilson[3], and Tereza Michalová[2]

[1] Crop Research Institute, Drnovská 507, Prague 6, Czech Republic

[2] Institute of Microbiology of the Czech Academy of Sciences, Vídeňská 1083, Prague 4, Czech Republic

[3] Department of Natural Resource Ecology and Management, Oklahoma State University, Stillwater, OK, USA

*Author for correspondence: Veronika Řezáčová; Tel: +420 241 062 382; Email: rezacova@vurv.cz; ORCID: 0000-0002-1749-0355

TABLE S1 Physicochemical properties of potting substrate mixed from previously sterilized components (soil : zeolite : sand, 10 : 45 : 45, v: v: v). pH (pH_{water}) was measured in aqueous soil suspension (1 : 2.5, w : v) after shaking for 1 h. Total phosphorus (P) was assessed colorimetrically following incineration of the substrate at 550°C and extraction with hot 14 M HNO_3. Water-extractable P was measured colorimetrically in aqueous soil extract (1 : 10, w : v) after shaking the suspension for 20 h and filtration through 0.2 μm membrane filter. Total nitrogen (N) and organic carbon (C) as well as isotopic composition of the C were assessed using a Flash EA 2000 elemental analyzer coupled with a Delta V Advantage isotope ratio mass spectrometer (Thermo Fisher Scientific, Waltham, MA, USA). Isotopic composition of the C is given with respect to the international Vienna Pee Dee Belemnite (VPDB) standard.

	mean value of 3 analytical reps
pH_{water}	8.90
Total P (mg kg^{-1})	46.5
Water-extractable P (mg kg^{-1})	2.95
Total N (%)	0.01
Total organic C (%)	0.22
$\delta^{13}C$ (vs. VPDB standard, in ‰)	−18.11

TABLE S2 Sequences of primers and hydrolysis probes (sequences (5′→3′)) used for the quantitative real-time PCR quantification of large ribosomal subunit gene copies of the different arbuscular mycorrhizal AM fungal taxa as described in Thonar et al. (2012).

	R. irregularis	*C. claroideum*	*F. mosseae*
Forward primer	TTCGGGTAATCAGCCTTTCG	GCGAGTGAAGAGGGAAGAG	GGAAACGATTGAAGTCAGTCATACCAA
Reverse primer	TCAGAGATCAGACAGGTAGCC	TTGAAAGCGTATCGTAGATGAAC	CGAAAAAGTACACCAAGAGATCCCAAT
Hydrolysis probe	TTAACCAACCACACGGGCAAGTACA	AACAGGACATCATAGAGGGTGACAATCCC	AGAGTTTCAAAGCCTTCGGATTCGC

TABLE S3 Percent of mycorrhizal fungal structures assessed microscopically in roots of weedy annual and perennial plant individuals planted in pairs. ‘Hyphae’ includes all AM fungal structures (hyphae, arbuscules, and vesicles) and can be considered total AM fungal colonization.

	Harvest 1			Harvest 2		
	Hyphae	Arbuscules	Vesicles	Hyphae	Arbuscules	Vesicles
E. annuus	43	33	3	41	14	3
C. biennis	35	31	2	43	21	8
C. canadensis	32	22	2	63	33	9
S. jacobaea	35	24	0	58	40	14

REFERENCES

Thonar C, Erb A, Jansa J (2012) Real-time PCR to quantify composition of arbuscular mycorrhizal fungal communities-marker design, verification, calibration and field validation. Molec Ecol Res 12, 219-232.

Table S4. Raw data table.

Article title: Arbuscular mycorrhiza can be disadvantageous for weedy annuals in competition with paired perennial plants

Authors: Veronika Řezáčová, Milan Řezáč, Gail W. T. Wilson and Tereza Michalová

Pot number	Weedy annual plant species	Harvest	Inoculation	Disturbance	Community composition	Shoot DW (g) of the left positioned (weedy annual or perennial) plant	Shoot DW (g) of the right positioned (always perennial) plant	Shoot P (mg P g^{-1} DW) of the left positioned (weedy annual or perennial) plant	Shoot P (mg P g^{-1} DW) of the right positioned (always perennial) plant	Shoot DW (g) of the nurse plants	LSU abundance of *Claroideoglomus claroideum* (gene copies mg^{-1} roots)	LSU abundance of *Glomus intraradices* (gene copies mg^{-1} roots)	LSU abundance of *Funneliformis mosseae* (gene copies mg^{-1} roots)
41	*Erigeron annuus*	1	M+	no	invasive-native	0.2735	0.1265	2.712107	3.554041	4.025	0	90 650	93 477
42	*Erigeron annuus*	1	M+	no	invasive-native	0.2145	0.1317	2.669231	2.603937	2.794	0	26 459	40 342
43	*Erigeron annuus*	1	M+	no	invasive-native	0.1134	0.188	3.519531	3.505511	3.778	0	8 487	90 372
44	*Erigeron annuus*	1	M+	no	invasive-native	0.0735	0.2431	3.496834	3.689861	4.077	0	536 720	136 225
45	*Erigeron annuus*	1	M+	no	invasive-native	0.0857	0.1705	4.10874	3.892642	4.571	0	133 482	501 799
46	*Erigeron annuus*	1	M+	no	native-native	0.2297	0.0897	3.996759	3.354869	4.476	0	555 681	178 342
47	*Erigeron annuus*	1	M+	no	native-native	0.2811	0.1951	3.630169	3.333513	3.843	113 578	79 974	95 346
48	*Erigeron annuus*	1	M+	no	native-native	0.1905	0.1863	3.189595	3.785063	4.341	0	1 400 272	137 954
49	*Erigeron annuus*	1	M+	no	native-native	0.3646	0.2292	3.099529	2.467562	3.523	49 832	155 642	97 322
50	*Erigeron annuus*	1	M+	no	native-native	0.1332	0.389	3.06431	3.032731	3.961	570 638	135 937	179 537
51	*Erigeron annuus*	1	M+	yes	invasive-native	0.101	0.0235	3.554207	3.033666	3.862	0	76 686	135 494
52	*Erigeron annuus*	1	M+	yes	invasive-native	0.1337	0.474	3.242282	2.938748	2.368	337 247	86 047	180 385
53	*Erigeron annuus*	1	M+	yes	invasive-native	0.2352	0.2342	2.805339	3.48665	4.079	39 006	53 069	215 995
54	*Erigeron annuus*	1	M+	yes	invasive-native	0.6335	0.2623	2.16617	2.41264	3.13	0	39 983	256 325
55	*Erigeron annuus*	1	M+	yes	invasive-native	0.1168	0.1878	3.185945	3.558845	3.907	0	426	187 808
56	*Erigeron annuus*	1	M+	yes	native-native	0.2163	0.2268	3.230986	3.395179	3.457	0	65 212	148 111
57	*Erigeron annuus*	1	M+	yes	native-native	0.2589	0.2276	3.44228	3.086518	3.968	0	68 201	156 327
58	*Erigeron annuus*	1	M+	yes	native-native	0.1175	0.3321	3.943578	3.581484	3.618	0	191 274	161 592
59	*Erigeron annuus*	1	M+	yes	native-native	0.2775	0.1916	3.584018	3.471704	2.84	0	103 465	224 555
60	*Erigeron annuus*	1	M+	yes	native-native	0.2325	0.1592	3.459274	2.972879	3.673	0	113 404	185 869
61	*Erigeron annuus*	1	M-	no	invasive-native	0.0937	0.0913	2.20075	1.497796	3.326			
62	*Erigeron annuus*	1	M-	no	invasive-native	0.0264	0.015	1.703844	1.004593	4.052			
63	*Erigeron annuus*	1	M-	no	invasive-native	0.0416	0.0657	1.986605	1.335597	4.102			
64	*Erigeron annuus*	1	M-	no	invasive-native	0.0183	0.0176	1.845561	1.265426	4.246			
65	*Erigeron annuus*	1	M-	no	invasive-native	0.0443	0.0413	2.078375	1.489801	3.401			
66	*Erigeron annuus*	1	M-	no	native-native	0.0292	0.0264	1.35001	1.344969	3.279			
67	*Erigeron annuus*	1	M-	no	native-native	0.0101	0.0475	1.535117	1.24103	3.559			
68	*Erigeron annuus*	1	M-	no	native-native	0.0891	0.0288	1.612426	1.251763	3.712			
69	*Erigeron annuus*	1	M-	no	native-native	0.0483	0.0342	2.374741	1.471291	3.905			
70	*Erigeron annuus*	1	M-	no	native-native	0.0805	0.0604	1.33981	1.128744	3.642			
71	*Erigeron annuus*	1	M-	yes	invasive-native	0.0664	0.0229	2.136626	1.19543	3.556			
72	*Erigeron annuus*	1	M-	yes	invasive-native	0.0326	0.0139	1.898259	0.98334	3.12			
73	*Erigeron annuus*	1	M-	yes	invasive-native	0.0314	0.0684	2.10714	1.762676	3.291			
74	*Erigeron annuus*	1	M-	yes	invasive-native	0.0277	0.0433	2.024219	1.069501	3.179			
75	*Erigeron annuus*	1	M-	yes	invasive-native	0.0808	0.0232	2.232613	1.274487	3.528			
76	*Erigeron annuus*	1	M-	yes	native-native	0.0102	0.0423	0.956864	1.106708	4.228			
77	*Erigeron annuus*	1	M-	yes	native-native	0.0203	0.0263	1.17673	1.337169	3.595			
78	*Erigeron annuus*	1	M-	yes	native-native	0.1015	0.0616	1.467139	1.360548	1.701			
79	*Erigeron annuus*	1	M-	yes	native-native	0.0538	0.0184	1.304209	1.423799	4			
80	*Erigeron annuus*	1	M-	yes	native-native	0.0385	0.0368	1.145496	1.123783	3.487			
81	*Conyza canadensis*	1	M+	no	invasive-native	0.13	0.0809	3.671726	3.874116	3.719	0	49 697	75 460
82	*Conyza canadensis*	1	M+	no	invasive-native	0.3025	0.0427	2.668083	2.59667	3.847	0	912 014	132 128
83	*Conyza canadensis*	1	M+	no	invasive-native	0.1549	0.1382	3.873251	4.114605	3.378	52 822	1 373 540	173 565

84	*Conyza canadensis*	1	M+	no	invasive-native	0.3281	0.2634	4.051473	3.806908	3.644	0	270 867	98 991
85	*Conyza canadensis*	1	M+	no	invasive-native	0.2723	0.276	3.792591	3.753746	3.368	0	234 874	166 256
86	*Conyza canadensis*	1	M+	no	native-native	0.1269	0.1071	3.203396	4.877336	4.364	0	299 365	338 359
87	*Conyza canadensis*	1	M+	no	native-native	0.1418	0.1189	3.932299	3.306413	4.095	0	315 110	182 022
88	*Conyza canadensis*	1	M+	no	native-native	0.1065	0.2358	3.876242	3.701621	3.759	0	35 356	97 555
89	*Conyza canadensis*	1	M+	no	native-native	0.0043	0.0628	0.573892	4.700578	4.537	0	45 343	97 191
90	*Conyza canadensis*	1	M+	no	native-native	0.0495	0.0201	3.529518	3.916443	4.268	0	184 223	200 586
91	*Conyza canadensis*	1	M+	yes	invasive-native	0.124	0.1628	3.737429	3.288731	3.481	70 371	11 614	71 844
92	*Conyza canadensis*	1	M+	yes	invasive-native	0.1396	0.2691	4.037404	3.674861	2.923	0	87 137	116 356
93	*Conyza canadensis*	1	M+	yes	invasive-native	0.1507	0.2126	3.602612	3.896468	3.141	0	62 037	165 565
94	*Conyza canadensis*	1	M+	yes	invasive-native	0.1553	0.2028	3.979586	4.26819	3.564	0	199 686	17 485
95	*Conyza canadensis*	1	M+	yes	invasive-native	0.115	0.2006	4.125284	3.772905	3.801	0	12 019	42 359
96	*Conyza canadensis*	1	M+	yes	native-native	0.1653	0.1152	3.51744	4.963468	3.797	0	54 833	215 999
97	*Conyza canadensis*	1	M+	yes	native-native	0.1492	0.0811	3.414354	3.425679	4.255	0	18 611	71 383
98	*Conyza canadensis*	1	M+	yes	native-native	0.1062	0.0649	1.683475	3.63887	3.544	0	4 891	143 976
99	*Conyza canadensis*	1	M+	yes	native-native	0.4312	0.1904	3.633645	3.321128	2.934	128 948	144 789	74 555
100	*Conyza canadensis*	1	M+	yes	native-native	0.0628	0.1428	3.859433	3.522999	4.225	0	5 050	167 403
101	*Conyza canadensis*	1	M-	no	invasive-native	0.0187	0.0175	2.227529	1.630511	4.342			
102	*Conyza canadensis*	1	M-	no	invasive-native	0.0256	0.0116	2.687262	1.998617	4.1			
103	*Conyza canadensis*	1	M-	no	invasive-native	0.0638	0.0378	2.488263	1.581478	3.22			
104	*Conyza canadensis*	1	M-	no	invasive-native	0.0775	0.1061	2.578167	1.577838	3.551			
105	*Conyza canadensis*	1	M-	no	invasive-native	0.0359	0.0927	3.350844	2.407468	4.191			
106	*Conyza canadensis*	1	M-	no	native-native	0.0149	0.038	2.160647	2.335554	4.263			
107	*Conyza canadensis*	1	M-	no	native-native	0.0074	0.0373	0.917958	1.946583	4.336			
108	*Conyza canadensis*	1	M-	no	native-native	0.0287	0.026	1.574216	1.683759	4.238			
109	*Conyza canadensis*	1	M-	no	native-native	0.0109	0.0294	0.970373	1.677313	3.952			
110	*Conyza canadensis*	1	M-	no	native-native	0.1413	0.3811	1.43573	0.820443	3.129			
111	*Conyza canadensis*	1	M-	yes	invasive-native	0.1271	0.2203	2.891018	2.589566	2.545			
112	*Conyza canadensis*	1	M-	yes	invasive-native	0.0707	0.1253	2.220297	1.956867	3.726			
113	*Conyza canadensis*	1	M-	yes	invasive-native								
114	*Conyza canadensis*	1	M-	yes	invasive-native	0.0483	0.0749	2.65484	1.7539	3.693			
115	*Conyza canadensis*	1	M-	yes	invasive-native	0.1132	0.1426	2.70185	1.705817	3.467			
116	*Conyza canadensis*	1	M-	yes	native-native	0.1176	0.0473	1.433779	1.583498	2.557			
117	*Conyza canadensis*	1	M-	yes	native-native	0.0369	0.0142	0.955239	1.156033	3.864			
118	*Conyza canadensis*	1	M-	yes	native-native	0.0416	0.2077	1.498587	1.974656	3.444			
119	*Conyza canadensis*	1	M-	yes	native-native	0.0802	0.024	1.952602	1.310416	4.075			
120	*Conyza canadensis*	1	M-	yes	native-native	0.021	0.0529	1.545604	2.044395	3.998			
161	*Erigeron annuus*	2	M+	no	invasive-native	1.2112	0.2996	2.081432	1.813933	2.926	0	12 364	30 614
162	*Erigeron annuus*	2	M+	no	invasive-native	1.0226	0.5169	2.138521	3.08802	4.599	0	21 832	36 849
163	*Erigeron annuus*	2	M+	no	invasive-native	1.0134	0.4388	2.438282	2.908796	4.763	0	37 716	68 727
164	*Erigeron annuus*	2	M+	no	invasive-native	1.2713	0.3776	2.012824	2.746567	3.623	0	35 051	54 455
165	*Erigeron annuus*	2	M+	no	invasive-native	1.0945	0.8867	1.77142	2.124855	3.144	0	20 448	30 241
166	*Erigeron annuus*	2	M+	no	native-native	0.3048	0.5617	2.719824	2.687415	4.442	0	82 222	70 988
167	*Erigeron annuus*	2	M+	no	native-native	0.7776	0.4673	2.304653	2.358622	3.314	0	14 535	36 875
168	*Erigeron annuus*	2	M+	no	native-native	0.2768	0.9332	2.115098	2.199467	4.169	0	24 895	71 182
169	*Erigeron annuus*	2	M+	no	native-native	0.4668	0.7719	2.197171	3.120077	3.713	0	59 678	60 061
170	*Erigeron annuus*	2	M+	no	native-native	0.8335	0.3768	2.230782	2.71529	3.888	0	42 341	51 022
171	*Erigeron annuus*	2	M+	yes	invasive-native	0.5204	0.7367	1.92703	3.012995	3.239	0	55 938	84 816
172	*Erigeron annuus*	2	M+	yes	invasive-native	1.5705	0.3827	1.663575	2.553258	3.731	0	0	29 890
173	*Erigeron annuus*	2	M+	yes	invasive-native	0.6125	0.5151	2.590872	3.150636	3.679	0	30 052	44 388
174	*Erigeron annuus*	2	M+	yes	invasive-native	0.9995	0.5301	2.082075	2.30641	4.021	0	16 353	66 534
175	*Erigeron annuus*	2	M+	yes	invasive-native	1.3949	0.2361	2.277741	2.627502	3.328	0	41 751	48 896
176	*Erigeron annuus*	2	M+	yes	native-native	0.5516	0.5399	2.72346	3.165667	3.734	0	32 184	116 667
177	*Erigeron annuus*	2	M+	yes	native-native	0.5498	0.5773	2.188942	2.267388	3.634	0	11 673	66 636
178	*Erigeron annuus*	2	M+	yes	native-native	0.5225	0.6156	2.06349	2.739519	4.436	0	18 469	59 973
179	*Erigeron annuus*	2	M+	yes	native-native	0.6544	0.2836	2.372089	2.340483	4.357	0	11 704	45 609
180	*Erigeron annuus*	2	M+	yes	native-native	0.7253	0.5937	2.359917	2.223708	4.038	0	0	62 376
181	*Erigeron annuus*	2	M-	no	invasive-native								
182	*Erigeron annuus*	2	M-	no	invasive-native	0.7711	0.1196	1.411831	0.791949	2.348			
183	*Erigeron annuus*	2	M-	no	invasive-native	0.5326	0.0579	1.413631	0.988711	3.981			
184	*Erigeron annuus*	2	M-	no	invasive-native	1.0342	0.033	1.126415	0.88271	3.138			
185	*Erigeron annuus*	2	M-	no	invasive-native	0.1819	0.0423	1.588409	1.207217	4.567			
186	*Erigeron annuus*	2	M-	no	native-native	0.0489	0.044	1.36581	0.944447	3.531			
187	*Erigeron annuus*	2	M-	no	native-native	0.3301	0.1893	1.239496	1.200762	3.583			
188	*Erigeron annuus*	2	M-	no	native-native	0.0781	0.0565	1.187256	1.097224	4.047			
189	*Erigeron annuus*	2	M-	no	native-native	0.1535	0.2152	1.195643	1.286075	3.455			
190	*Erigeron annuus*	2	M-	no	native-native	0.0892	0.1153	1.080789	1.226781	3.575			
191	*Erigeron annuus*	2	M-	yes	invasive-native	0.3861	0.0391	1.490433	0.945834	3.891			
192	*Erigeron annuus*	2	M-	yes	invasive-native	0.702	0.0981	1.343678	1.441076	2.974			
193	*Erigeron annuus*	2	M-	yes	invasive-native	0.3816	0.086	1.167331	1.095921	4.025			
194	*Erigeron annuus*	2	M-	yes	invasive-native	0.3707	0.0354	1.468821	1.016759	3.854			

195	*Erigeron annuus*	2	M-	yes	invasive-native	0.2508	0.1024	1.415623	1.105941	3.633			
196	*Erigeron annuus*	2	M-	yes	native-native	0.0638	0.1381	1.252308	1.293121	3.961			
197	*Erigeron annuus*	2	M-	yes	native-native	0.0348	0.038	1.203878	1.324285	3.872			
198	*Erigeron annuus*	2	M-	yes	native-native	0.0363	0.099	1.311379	1.46736	3.057			
199	*Erigeron annuus*	2	M-	yes	native-native	0.0736	0.1621	2.031128	1.277444	3.211			
200	*Erigeron annuus*	2	M-	yes	native-native	0.0331	0.1254	1.175256	1.09097	3.958			
201	*Conyza canadensis*	2	M+	no	invasive-native	1.1746	0.286	3.549543	2.97676	3.708	0	12 976	11 932
202	*Conyza canadensis*	2	M+	no	invasive-native	0.8894	0.1	4.721827	3.120923	3.444	0	23 491	1 173
203	*Conyza canadensis*	2	M+	no	invasive-native	0.6501	0.7571	3.833591	3.030947	3.842	0	5 908	5 469
204	*Conyza canadensis*	2	M+	no	invasive-native	1.1076	0.3735	3.683152	2.869247	4.315	0	47 409	3 294
205	*Conyza canadensis*	2	M+	no	invasive-native	0.7176	0.5551	3.547005	3.522891	3.682	0	29 173	4 307
206	*Conyza canadensis*	2	M+	no	native-native	0.3854	0.7176	2.485026	2.615758	4.602	0	7 456	5 724
207	*Conyza canadensis*	2	M+	no	native-native	0.2725	0.2073	4.282875	4.229083	4.671	0	119 279	24 060
208	*Conyza canadensis*	2	M+	no	native-native	0.421	1.1746	2.761381	3.146092	3.739	0	42 818	5 644
209	*Conyza canadensis*	2	M+	no	native-native	0.7462	0.4831	3.276263	2.837506	3.992	0	42 232	7 980
210	*Conyza canadensis*	2	M+	no	native-native	0.2976	0.1776	3.950105	4.798189	4.015	0	86 202	30 932
211	*Conyza canadensis*	2	M+	yes	invasive-native	0.5144	0.4416	3.953527	3.107476	3.953	0	44 437	9 468
212	*Conyza canadensis*	2	M+	yes	invasive-native	1.165	0.8368	3.65702	3.066636	3.061	0	5 966	4 540
213	*Conyza canadensis*	2	M+	yes	invasive-native	0.2756	1.1945	3.013185	3.505201	4.2571	0	3 408	1 109
214	*Conyza canadensis*	2	M+	yes	invasive-native	1.197	0.1656	3.439605	2.772176	4.98	0	1 767	828
215	*Conyza canadensis*	2	M+	yes	invasive-native	0.948	0.3538	4.041952	3.648709	3.73	0	2 714	1 336
216	*Conyza canadensis*	2	M+	yes	native-native	0.7618	0.4341	3.786004	3.158816	3.755	52 198	8 950	3 158
217	*Conyza canadensis*	2	M+	yes	native-native	0.2867	1.2631	2.199028	2.63963	3.452	0	2 852	3 851
218	*Conyza canadensis*	2	M+	yes	native-native	0.6839	0.8568	3.115881	2.993159	2.921	0	1 989	1 643
219	*Conyza canadensis*	2	M+	yes	native-native	1.1548	0.2615	3.219743	2.878766	3.37	0	0	2 089
220	*Conyza canadensis*	2	M+	yes	native-native	0.698	0.602	3.486029	3.078144	3.633	0	1 195	5 212
221	*Conyza canadensis*	2	M-	no	invasive-native	0.1722	0.0669	2.217939	1.474909	4.238			
222	*Conyza canadensis*	2	M-	no	invasive-native	0.493	0.1199	1.869343	1.380678	4.067			
223	*Conyza canadensis*	2	M-	no	invasive-native	0.3746	0.2948	1.912092	1.646943	3.297			
224	*Conyza canadensis*	2	M-	no	invasive-native	0.3324	0.1661	1.851376	1.653213	3.318			
225	*Conyza canadensis*	2	M-	no	invasive-native	0.569	0.1108	2.005361	1.273324	3.315			
226	*Conyza canadensis*	2	M-	no	native-native	0.0185	0.1045	0.961491	1.891283	3.494			
227	*Conyza canadensis*	2	M-	no	native-native	0.031	0.0939	1.474746	1.57446	3.752			
228	*Conyza canadensis*	2	M-	no	native-native								
229	*Conyza canadensis*	2	M-	no	native-native	0.0393	0.0101	1.600282	1.430726	4.301			
230	*Conyza canadensis*	2	M-	no	native-native	0.2634	0.1318	2.466534	1.96892	3.877			
231	*Conyza canadensis*	2	M-	yes	invasive-native	0.5909	0.0916	2.416814	1.576035	2.948			
232	*Conyza canadensis*	2	M-	yes	invasive-native	0.7573	0.0205	1.666698	1.320398	2.656			
233	*Conyza canadensis*	2	M-	yes	invasive-native	0.4026	0.0397	2.069071	1.378656	3.516			
234	*Conyza canadensis*	2	M-	yes	invasive-native	0.6511	0.7532	1.636929	1.856734	3.084			
235	*Conyza canadensis*	2	M-	yes	invasive-native	0.6552	0.8923	1.613088	1.555003	3.332			
236	*Conyza canadensis*	2	M-	yes	native-native	0.2565	1.1736	1.412917	3.135743	3.38			
237	*Conyza canadensis*	2	M-	yes	native-native	0.013	0.1588	2.313875	1.998554	3.117			
238	*Conyza canadensis*	2	M-	yes	native-native	0.3254	0.1375	1.804563	2.004599	3.607			
239	*Conyza canadensis*	2	M-	yes	native-native	0.0341	0.3729	1.470652	2.342295	3.91			
240	*Conyza canadensis*	2	M-	yes	native-native	0.1181	0.0256	1.688656	1.534345	3.697			
